# Detection of *CCNE1/URI* (19q12) amplification by *in situ* hybridisation is common in high grade and type II endometrial cancer

**DOI:** 10.18632/oncotarget.11605

**Published:** 2016-08-25

**Authors:** Aurelia Noske, Simone Brandt, Nadejda Valtcheva, Ulrich Wagner, Qing Zhong, Elisa Bellini, Daniel Fink, Ellen C. Obermann, Holger Moch, Peter J. Wild

**Affiliations:** ^1^ Institute of Surgical Pathology, University Hospital Zurich, Zurich, Switzerland; ^2^ Department of Gynaecology, University Hospital Zurich, Zurich, Switzerland; ^3^ Institute of Pathology, University Hospital Basel, Basel, Switzerland

**Keywords:** endometrial cancer, in situ hybridisation, 19q12

## Abstract

One TCGA subgroup of endometrial cancer (EC) is characterised by extensive genomic DNA copy number alterations. *CCNE1* located at 19q12 is frequently amplified in EC and a target for anti-cancer therapy. The relevance of *URI*, also located at 19q12, is unknown. To evaluate the prevalence of 19q12 (*CCNE1/URI*) in EC, we investigated different histologic types by *in situ* hybridisation (ISH) and copy number assay. We applied a previously established 19q12 ISH for the detection of *CCNE1/URI* copy numbers in EC (n = 270) using conventional bright field microscopy. In a subset (n = 21), 19q12 amplification status was validated by OncoScan assay. Manual ISH was controlled by a recently developed computational ISHProfiler algorithm. Associations of 19q12 status with Cyclin E1, URI and p53 expression, and clinico-pathological parameters were tested.

Amplification of 19q12 (*CCNE1/URI*) was found in 10.4% (28/270) and was significantly associated with type II EC (high grade and non-endometrioid; *p* < 0.0001), advanced FIGO stage (*p* = 0.001), high Cyclin E1 expression (*p* = 0.008) and aberrant p53 expression (*p* = 0.04). 19q12 ISH data were confirmed by OncoScan and computational ISHProfiler techniques. The 19q12 *in situ* hybridisation is a feasible and robust biomarker assay in molecular pathology. Amplification of *CCNE1/URI* predominantly occurred in type II endometrial cancer. Prospective clinical trials are warranted to assess the utility of combined 19q12 amplification and Cyclin E1/URI protein expression analysis for the prediction of therapeutic response to chemotherapy and/or cyclin-dependent kinase inhibitors in patients with endometrial cancer.

## INTRODUCTION

Endometrial cancer (EC) is traditionally subdivided into types I and II based on histological type, tumour differentiation and clinical features [1]. Type I includes low-grade endometrioid EC and variants, showing a favourable prognosis. Conversely, type II tumours comprise high-grade and non-endometrioid cancers (serous and clear cell) that are less frequent (10–20%) but more aggressive with characteristic poor outcomes. At molecular levels, types I and II EC share several genetic abnormalities (frequent *PTEN*, *PIK3CA* and *KRAS* mutations). Contrary to type I, most type II carcinomas exhibit a high frequency of *TP53* mutations. The Cancer Genome Atlas (TCGA) analysis of endometrial carcinomas revealed four genomic groups; among them was the ‘copy number (CN) high’ group termed ‘serous-like’, comprising serous, mixed and high-grade endometrioid ECs showing frequent *TP53* mutations, *CCNE1* (19q12) amplification, rare microsatellite instability and fewer *PTEN* mutations than other ECs [2].

Genomic amplification of *CCNE1* and increased expression of the encoded protein Cyclin E1 has been previously shown in serous EC [3, 4]. *CCNE1* drives the genesis of uterine serous carcinomas mainly by activating cell-cycle progression through CDK2 activation, Rb phosphorylation and E2F-1-mediated transcription [3]. However, the 19q12 amplicon comprises several genes along with *CCNE1*, including *URI (C19Orf2, RPB5-mediating protein), C19Orf12, POP4* and *PLEKHF1*. We and others recently observed *URI* amplification in carcinomas of the ovary [5–7] and endometrium [6]. The URI protein belongs to the prefoldin family of molecular chaperones involved in translational control-related pathways [8]. URI overexpression in ovarian cancer cells promotes cell survival and contributes to the oncogenic effect of 19q12 amplification [6].

Patients with type II EC have a high relapse risk and poor prognosis, particularly those belonging to the ‘copy number high’ EC genomic subgroup [2]. Guidelines recommend treatment by surgery, adjuvant radiation and chemotherapy in patients with high-grade and/or advanced EC. Currently, the long-term effectiveness of chemotherapy (usually platin–taxane-based) is uncertain [9]. Although therapy results in an initial complete response, resistance development is a major problem [10]. Chemoresistance in high-grade serous ovarian carcinomas is attributed to amplified *CCNE1* [11, 12]. Since endometrial and ovarian carcinomas show a genomic relationship, the 19q12 amplification status may also have an impact on EC therapy. Additionally, while several targeted drug clinical trials were completed, no targeted therapies were approved for EC. Novel drugs and combinations are constantly being introduced, but there are few predictive markers and tests for patient selection. The 19q12 amplicon is a potential predictive marker for response to conventional chemotherapy and CDK inhibitors [12–14]. Treatment decisions could potentially depend on the 19q12 (*CCNE1/URI*) copy number status in combination with Cyclin E1 and/or URI protein expression.

We evaluated the prevalence and degree of 19q12 (including *CCNE1* and *URI*) amplification in a large group of archived EC samples using a recently established chromogenic *in situ* hybridisation (ISH) assay for automated 19q12 detection with immunohistochemical Cyclin E1 and URI protein expression [7]. To validate the 19q12 ISH data, we analysed a subset of samples for CN changes using the Affymetrix OncoScan assay. Additionally, manually assessed 19q12 ISH was independently scored by a computational ISHProfiler algorithm.

## RESULTS

### EC sample characteristics

Totally, 436 endometrial carcinomas were studied. Endometrioid carcinoma (and variants) was the most common subtype (361, 83.8%). Less frequent was the non-endometrioid subtype (61, 12.6%). In 16 samples (3.6%), the histological type could not be determined. According to the traditional histological EC categorisation, 310 (71.1%) were type I (low-grade endometrioid and mucinous) and 102 were type II (high-grade endometrioid and non-endometrioid). Subtyping was impossible in 5.5% samples. Most cases were diagnosed in an early FIGO stage (58.9%). Characteristics are listed in Table 1.

**Table 1:** Pathological characteristics of EC samples (n = 436)

Characteristics	n (%)
**Histological type**	
endometrioid	359 (83.3)
mucinous	2 (0.5)
serous	22 (5.1)
clear cell	17 (3.9)
carcinosarcoma (MMMT)	12 (2.8)
undifferentiated	8 (1.9)
unclassifiable	16 (3.6)
**Type I & II category**	
Type I	310 (71.1)
Type II	102 (23.4)
unclassifiable	24 (5.5)
**FIGO stage**	
early	257 (58.9)
late	84 (19.3)
missing	95 (21.8)

MMMT: malignant mixed Müllerian tumour

### 19q12 amplification occurs more frequently in type II EC

**Figure 1:**
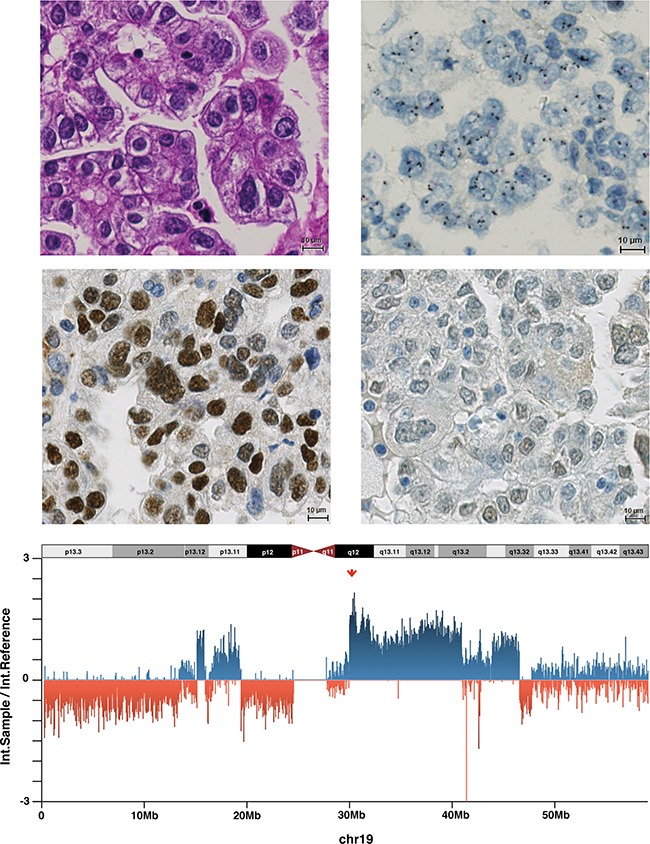
Endometrial cancer (Case 33) with 19q12 amplification, high Cyclin E1 expression, URI negativity and high 19q12 copy number **A**. Endometrial carcinoma of the clear cell type belonging to type II (HE staining). **B**. Amplification of 19q12 in a dual-colour ISH (19q12 black signals, reference red signals). **C**. High Cyclin E1 expression in the tumour cell nuclei. **D**. Absence of URI expression. **E**. Intensity ratios (intensity sample/intensity reference) for all chromosome 19-related probes of the OncoScan assay. High copy numbers at 19q12 (*CCNE1/URI*) locus are indicated by the red arrow.

The 19q12 ISH assay was applied to two TMA cohorts (Basel and Zurich-TMA), both including different histological EC subtypes. Tissue cores containing at least 50 tumour cells were evaluated to calculate CN. Due to tumour tissue paucity, crush artefacts or weak ISH signals, not all tissue cores were amenable to analysis. Assay interpretation was possible for 270 tissue samples and was performed by a board certified pathologist (A.N.). 19q12 enumeration and chromosome 19 signals were manually completed for 50 tumour nuclei/sample with amplification defined as 19q12/chr19 ratio ≥2.0 [7, 15]. Using this cut-off, copy number alterations were found in 28/270 carcinomas (10.4%). 19q12 amplification was significantly associated with type II EC (comprising high-grade and non-endometrioid cancer) and advanced FIGO stage (Table 2, Figure 1A-1B). In univariate Kaplan–Meier analysis, patients with 19q12 amplified carcinomas tended to have worse overall survival, but this trend did not reach statistical significance (log-rank test, *p* = 0.075).

**Table 2:** Associations between pathological parameters and 19q12 amplification, Cyclin E1, and URI protein expression

	19q12 Amplificationn (%)	p-value*	Cyclin E1 highn (%)	p-value*	URI highn (%)	p-value*
**Histology**^1^		<0.0001		<0.0001		0.009
endometrioid	13/230 (5.6)		162/350 (46.2)		38/353 (10.7)	
non-endometrioid	15/37 (40.5)		44/58 (75.8)		14/58 (24.1)	
**Tumour type**^2^		<0.0001		<0.001		0.010
type I	8/189 (4.2)		137/300 (45.6)		31/302 (10.2)	
type II	19/70 (27.1)		65/100 (65)		21/101 (20.8)	
**FIGO stage**		0.002		0.31		0.83
early	8/148 (5.4)		126/244 (51.6)		24/248 (9.6)	
late	11/51 (21.5)		49/84 (58.3)		7/84 (8.3)	

* Fisher's Exact Test

1 non-endometrioid carcinomas (including serous, clear cell, undifferentiated, carcinosarcoma)

2 type I endometrioid (low grade, G1-2) and mucinous; type II endometrioid (high grade, G3) and non-endometrioid histology

### Cyclin E1 and URI protein expression

Cyclin E1 immunohistochemical analysis was possible in 412 carcinomas. The H-score (range 0–300) and afterwards the median H-score (140) was calculated to distinguish low from high Cyclin E1 expression. High expression levels were observed in 208/412 cases (50.5%). Increased Cyclin E1 expression was significantly more frequent in type II and non-endometrioid EC (Table 2, Figure 1C). In univariate Kaplan–Meier analysis and univariate Cox regression analysis, patients with high Cyclin E1 expressing carcinomas had significantly shorter overall survival time than patients with low Cyclin E1 expression (log-rank test, *p* = 0.017; HR = 1.59, 95%CI, 1.09–2.3). However, an independent prognostic value of Cyclin E1 overexpression was not confirmed by multivariate COX regression analysis adjusted for other parameters (patient age, tumour subtype and FIGO stage; *p* = 0.079).

Evaluation of URI protein expression by immunohistochemistry was possible in 416 carcinomas. URI expression was exclusively detected in cytoplasm. For statistics, we categorised the expression as recently described [7]. Moderate and strong cytoplasmic staining (defined as URI-positive) was observed in 12.5% of the carcinomas (52/416) but neither associated with pathological features (Table 2, Figure 1D) nor overall survival (log-rank test, *p* = 0.68).

### Association of 19q12 amplification status with CCNE1 and URI expression

As the 19q12 ISH assay covers both *CCNE1* and *URI*, we tested the associations of 19q12 amplification status with Cyclin E1 and URI protein expression levels (Table 3). 19q12 amplification was significantly associated with high Cyclin E1 expression (*p* = 0.008, Fisher's exact test). However, there were cases with high Cyclin E1 expression without 19q12 amplification and 19q12-amplified tumours with low Cyclin E1 expression. No association between 19q12 amplification status and URI expression was found (*p* = 0.593, Fisher's exact test). Cyclin E1 expression was strongly proportionate to URI expression (*p* = 0.026, Fisher's exact test).

**Table 3:** Associations between 19q12 amplification and Cyclin E1, URI and p53 immunohistochemical expression levels

	all cases	19q12 amplification n (%)	p-value*
**Cyclin E1**			0.008
low expression	144	8 (5.5)	
high expression	121	19 (15.7)	
**URI**			0.593
low expression	221	22 (9.9)	
high expression	45	6 (13.3)	
**p53**			0.026
wild type pattern	127	8 (6.2)	
aberrant pattern	128	20 (15.6)	

* Fisher's Exact Test

The above analysis used the median H-score to define two broad expression classes; many similar individual values (near the median) were segregated. To more precisely examine potential associations between immunohistochemistry (IHC) and ISH findings, we performed a correlation analysis using pure immunohistochemistry characteristics as staining intensity or percentage of positive cells. However, as indicated in the correlation plot (Supplementary Figure S1), the relationships between 19q12 ISH and both intensity and fraction of Cyclin E1 and URI positive cells were low.

### Aberrant p53 immunohistochemistry is related to 19q12 amplification

Since *TP53* mutations are common in EC type II, we examined the associations of p53 immunohistochemical staining pattern with 19q12 amplification status, Cyclin E1 expression, and URI expression. According to the WHO classification 2014, aberrant p53 staining (diffuse and strong positivity in >75% tumour cells or complete lack of staining) correlates with a *TP53* mutation, while variable staining intensity in less than 75% of the tumour cells is related to wild-type *TP53* (WHO classification 2014). Using this classification, we observed a significant association between aberrant *TP53* expression and 19q12 amplification status (Table 3). High Cyclin E1 expression was significantly more common in EC with aberrant p53 expression (*p* = 0.033, Fisher's exact test), while URI expression was unrelated to p53 expression (*p* = 0.53, Fisher's exact test).

### 19q12 amplification confirmation by OncoScan assay and computational methods

For ISH assay validation, we conducted the OncoScan assay in a subset of 21 EC samples with known histological grade and ISH-determined CN. Totally, 34–214 ng dsDNA was obtained from these samples. Although Affymetrix recommends 80 ng genomic dsDNA as the input for the OncoScan FFPE Assay, all 21 samples fulfilled the quality control criteria for amplified and cleaved probes. The parameters measured from the signal intensities, such as MAPD and ndSNP quality control, were also considered acceptable for determining CN.

**Figure 2:**
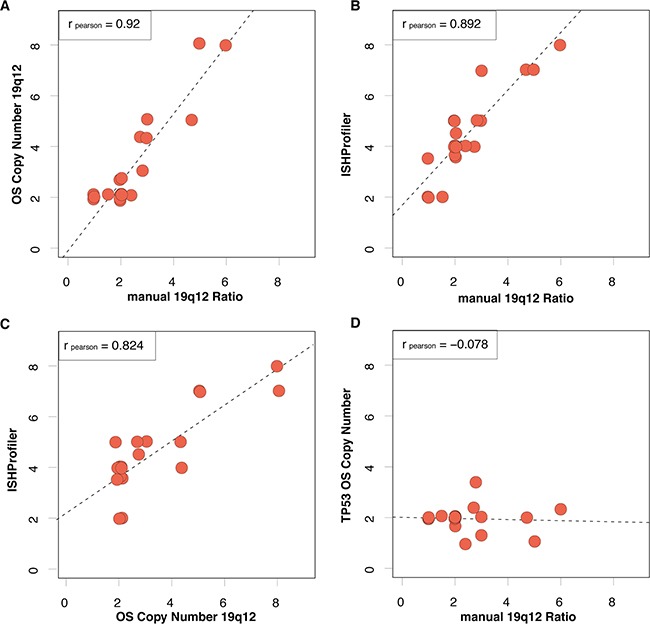
Correlations among 19q12 copy numbers as measured manually by ISH, OncoScan (OS) or by ISHProfiler algorithm **A**. Pearson correlation of manually assessed 19q12 ISH ratio (cut-off ≥ 2) and copy number at the 19q12 locus by OncoScan. Data points with exactly the same x- and y-values are separated by data jittering. The trend between 19q12 ratio and CN based on linear regression is plotted as a dashed line. **B**. The same correlation for manually assessed 19q12 ISH ratio and ISHProfiler ratio. **C**. The same correlation for ISHProfiler ratio and OS copy number at the 19q12 locus. **D**. In contrast, no correlation for the manually assessed ISH ratio and OS copy number at the *TP53* locus.

Consistent with the ISH assay, CNVs (measured by OncoScan) were more frequent in high-grade and non-endometrioid EC compared to low-grade endometrioid EC (Supplementary Figure S2). We observed 19q12 CN gains in 9/21 EC samples using the OncoScan FFPE Assay (Table 4), high CN gain (CN > 3) in six cases (shown in Figure 1E) and 2 < CN ≤ 3 in three cases. Additionally to *CCNE1* and *URI*, other 19q12 locus members, including *POP4 and PLEKHF1*, showed the same CN levels.

**Table 4:** Correspondence of 19q12 amplification status determined by *in situ* hybridisation (ISH) and OncoScan assay

			19q12 amplicon OncoScan assay			
Case	EC subtype	19q12 ISH amplification	CCNE1	URI1	POP4	PLEKHF1
026	serous	high	High CN gain	High CN gain	High CN gain	High CN gain
033	clear cell	high	High CN gain	High CN gain	High CN gain	High CN gain
042	hg endometrioid	high	High CN gain	High CN gain	High CN gain	High CN gain
040	serous	high	High CN gain	High CN gain	High CN gain	High CN gain
034	hg endometrioid	high	High CN gain	High CN gain	High CN gain	High CN gain
027	hg endometrioid	low	High CN gain	High CN gain	High CN gain	High CN gain
046	serous	low	CN gain	CN gain	CN gain	CN gain
029	carcinosarcoma	low	CN gain	CN gain	CN gain	CN gain
045	serous	low	CN gain	CN gain	CN gain	CN gain
028	hg endometrioid	low	-	-	-	-
031	lg endometrioid	low	-	-	-	-
035	lg endometrioid	low	-	-	-	-
036	lg endometrioid	low	-	-	-	-
037	serous	low	-	-	-	-
038	hg endometrioid	low	-	-	-	-
039	lg endometrioid	low	-	-	-	-
041	lg endometrioid	low	-	-	-	-
030	lg endometrioid	no	-	-	-	-
047	lg endometrioid	no	-	-	-	-
048	lg endometrioid	no	-	-	-	-
043	lg endometrioid	no	-	-	-	-

lg: low grade; hg: high grade

The OncoScan platform detected high 19q12 CN in all five EC samples determined as high amplification by ISH (19q12/chr19 ratio ≥3; Table 4). One high-grade endometrioid EC sample with apparent low 19q12 amplification by ISH (Case 27) showed high CN by OncoScan. Conversely, of the 12 EC samples with low amplification determined by 19q12 ISH (19q12/chr19 ratio ≥2–3), four showed CN gain and eight showed stable CN in the OncoScan assay. Of these 12 EC samples with low 19q12 amplification determined by ISH, only high-grade, non-endometrioid EC (3/6) but no low-grade endometrioid EC (0/5) samples displayed CN changes at 19q12 by the OncoScan assay. Four low-grade endometrioid EC samples showed no CNVs at 19q12 in either of the two assays.

We frequently observed *TP53* alterations in ECs with 19q12 CN gain compared to ECs without 19q12 CN alterations. 19q12 amplification/CN gain was related to *TP53* loss of heterozygosity (LOH) and an aberrant p53 immunohistochemistry pattern suggesting mutation (but not deletion) (Table 5).

**Table 5:** Contribution of 19q12 amplification status to *TP53* alterations

Case	EC subtype	19q12 ISH amplification	OncoScan 19q12 amplicon	OncoScanTP53	p53 IHC positive cells	p53 IHC pattern
026	serous	high	High CN gain	LOH	0%	aberrant
033	clear cell	high	High CN gain	LOH, CN loss	95%	aberrant
042	hg endometrioid	high	High CN gain	CN gain	30%	wt
040	serous	high	High CN gain	LOH	100%	aberrant
034	hg endometrioid	high	High CN gain	LOH, CN loss	100%	aberrant
027	hg endometrioid	low	High CN gain	LOH/CN gain	0%	aberrant
046	serous	low	CN gain	LOH/CN gain	0%	aberrant
029	carcinosarcoma	low	CN gain	LOH	100%	aberrant
045	serous	low	CN gain	LOH	100%	aberrant
028	hg endometrioid	low	x	LOH	10%	wt
031	lg endometrioid	low	x	x	0%	aberrant
035	lg endometrioid	low	x	x	30%	wt
036	lg endometrioid	low	x	x	0%	aberrrant
037	serous	low	x	LOH	100%	aberrrant
038	hg endometrioid	low	x	x	20%	wt
039	lg endometrioid	low	x	x	15%	wt
041	lg endometrioid	low	x	x	5%	wt
030	lg endometrioid	no	x	x	0%	aberrrant
047	lg endometrioid	no	x	x	5%	wt
048	lg endometrioid	no	x	x	5%	wt
043	lg endometrioid	no	x	x	n.a.	n.a.

lg: low grade; hg: high grade; LOH: loss of heterozygosity

Statistical analysis revealed a high concordance between manual assessment of 19q12 ISH and OncoScan-based CN analysis (r = 0.92, Figure 2A). We further used the ISHProfiler algorithm in the same subset of 21 ECs [16]. Manually evaluated 19q12 ISH CN highly correlated with computational ISHProfiler CN (r = 0.89, Figure 2B). A similar correlation was observed between 19q12 CN by OncoScan assay and by ISHProfiler (r = 0.82, Figure 2C). In terms of a negative control, we demonstrate a no existing relation between manual 19q12 ISH CN and *TP53* CN (r = −0.1012, Figure 2D).

## DISCUSSION

Current adjuvant treatment for high-grade and advanced EC is based on radiation and/or chemotherapy. However, the effectiveness of chemotherapy is uncertain and approved targeted therapies and/or predictive tests for patient selection are not available. To guide treatment decisions, detailed knowledge of oncogenic drivers is required. TCGA data has shown that solid carcinomas such as high-grade serous ovarian carcinomas and serous-like endometrial carcinomas are frequently driven by CN alterations rather than by somatic mutations [2, 17]. The ‘copy number high’ TCGA subgroup of EC is characterised by amplification of *MYC*, *ERBB2*, *CCNE1*, *FGFR3* and *SOX17*. We therefore investigated the 19q12 amplicon, including the oncogenic drivers *CCNE1* and *URI*, in a large EC cohort including different histological types. We observed 19q12 (*CCNE1/URI*) amplification in 10% of the entire EC group, most frequently in advanced stage non-endometrioid and high-grade endometrioid carcinomas (type II). This is consistent with TCGA results demonstrating significant *CCNE1* (19q12) amplification in nearly 22% of the ‘serous-like/cluster 4’ tumours [2]. Using SNP arrays, Kuhn and colleagues recently reported *CCNE1* amplification in 26.1% uterine serous carcinomas [3]. They also found amplification in up to 45% (20 out of 44) of the serous carcinomas using FISH [4]. If we consider only the high-grade and non-endometrioid ECs in our cohort, the amplified cases constitute 40.5% (15/37).

Our recently established 19q12 ISH assay covers not only *CCNE1* but also *URI*. These genes are located in close proximity, but it is unclear if *URI* is also an oncogenic driver of this amplicon. We and others have recently shown that *URI* (*C19orf2*) is amplified in a subset of ovarian carcinomas and ovarian cancer cell lines [5, 6], suggesting that *CCNE1* may not be the exclusive driver at 19q12. The exact role of *URI* in EC is largely unknown but we previously demonstrated *URI* amplification by FISH in 4.6% (5/108) of these carcinomas [6].

To validate 19q12 amplification status as assessed by ISH and to explore the 19q12 locus, we subjected 21 ISH-characterised EC samples to OncoScan assay, which enables accurate whole genome CN estimation. As expected, we observed a different profile and frequency of DNA CN alterations among histological EC subtypes. Consistent with TCGA findings, high-grade endometrioid and serous carcinomas were characterised by extensive CN changes compared to low-grade endometrioid EC. This assay further confirmed our manual 19q12 ISH assessment. All carcinomas with high 19q12 amplification levels as detected by ISH (19q12/chr19 ratio ≥ 3) displayed high CN gain by OncoScan. However, a few carcinomas with low amplification as assessed by 19q12 ISH (19q12/chr19 ratio ≥2 but <3) showed high CN gain by OncoScan. Of note, only high-grade EC (4/7) with low level 19q12 amplification by ISH showed CN gain by OncoScan, suggesting ISH may have lower sensitivity for CN gain detection, at least under certain conditions. The evaluation and stratification of 19q12 ISH was performed according to our recent study based on previously published recommendations [4, 7]. Alternatively, OncoScan technology allows for more detailed resolution and subdivision and thus may be superior for low CN gain. In this case, the clinical significance of 19q12/chr19 ratio ≥2 but <3 by ISH should be re-assessed. It must be noted, however, that manual evaluation of ISH staining was conducted on tissue microarrays with limited amounts of tumour tissue. Analysis of whole tissue sections would likely yield more accurate CN estimates and better agreement with OncoScan.

The OncoScan assay also revealed additional genomic events at the 19q12 amplicon. Concurrent CN gains were observed not only for *CCNE1* and *URI* but for six additional genes in close vicinity: C19Orf12, *POP4, PLEKHF1, VSTM2B, UQCRFS1* and *LOC284395*. *CCNE1* was once considered the exclusive oncogenic driver within the 19q12 amplicon [18], but the present study and recent studies on ovarian and breast carcinomas have identified co-amplified genes [6, 19] that may also contribute to tumour aggression or treatment resistance [6, 7]. In our recent study, 75% *URI*-amplified epithelial ovarian carcinomas (EOCs) showed co-amplification of *CCNE1* (nine out of 12), suggesting sole amplification of *URI* occurs in 25% EOC cases. On a functional level, *URI* but not *CCNE1* enhanced the viability of ovarian cancer cell lines in a high-throughput siRNA screen [5]. In another study, *CCNE1* was amplified in only five of 16 primary breast carcinomas harbouring amplification of the 19q12 locus [19]. It was further demonstrated by microarray gene expression analysis that *CCNE1*, *C19Orf2 (URI)*, *C19Orf12*, *POP4*, *PLEKHF1* and *UQCRFS1* were significantly overexpressed when amplified and that silencing of *CCNE1, POP4* and *PLEKHF1* reduced cell viability. Accordingly, *CCNE1* is not the only driver of the 19q12 amplicon. Therefore, other 19q12 amplicon genes should be included in amplification assays.

The TCGA datasets revealed a genomic relationship between endometrial serous-like and high-grade serous ovarian carcinomas (HGSOC) [2]. Both are characterised by high CNVs and frequent *TP53* mutations. Similar to high-grade and non-endometrioid EC, amplification of the 19q12 (*CCNE1*) locus has been reported in up to 30% [17, 20–22] and *URI* in 10%–15% HGSOCs [6, 7]. In both EC and HGSOC, we found an association between 19q12 amplification (*CCNE1/URI*) and loss or increased p53 immunohistochemical expression, suggestive of *TP53* mutation [7]. This observation is supported by the subset of EC samples subjected to OncoScan showing a relationship between 19q12 amplification/CN gain and *TP53* LOH. Alternatively, Kuhn et al. found no significant correlation between *CCNE1* gene amplification assessed by FISH and *TP53* mutation in uterine serous carcinomas [4].

19q12 amplification status is a critical chemotherapy response determinant and therapeutic target in other cancer therapies. Primary treatment failure (platinum-based chemotherapy) in ovarian cancer was attributed to *CCNE1* amplification [11] and partly to an intact BRCA1/2 pathway [23]. *URI* amplification may also mediate resistance to cisplatin in ovarian cancer cells [6]. *CCNE1-*amplified ovarian cancer cells were more sensitive to cell cycle arrest, growth inhibition and apoptosis induction by *CCNE1* siRNA [21]. Additionally, *CCNE1* function can be modulated via its partner kinase *CDK2* [24]. *CCNE1-*amplified breast cancer cells were sensitive to *CDK2* inhibitors, resulting in reduced cancer cell survival [19]. The relevance of 19q12 amplicon/*CDK2* as a predictive marker for chemotherapy response and anti-19q12/*CDK2*-targeted therapies in EC remains unknown. Clinical trials are needed to evaluate whether 19q12 (*CCNE1/URI)* amplicon status can help identify patients most likely to respond to standard treatment or benefit from therapeutic approaches targeting cell cycle checkpoints. Our novel automated 19q12 ISH is a feasible biomarker assay and could be applied for routine diagnosis. Whether a combination of both 19q12 ISH assay and protein expression analysis of Cyclin E1 and URI by IHC is suitable, similar to HER2 assessment in breast cancer, needs further investigation.

## MATERIALS AND METHODS

### EC sample cohort

Primary EC samples were collected between 1985 and 2005, fixed in 4% neutral buffered formaldehyde, embedded in paraffin and stored in the archives of the Institutes of Pathology, University of Basel and University of Zurich (Switzerland) [25]. Routine haematoxylin and eosin sections were processed for additional histopathological evaluation. Histological subtype and grade were reviewed and defined (WHO classification 2014). Tumour stage was assessed according to International Federation of Gynaecology and Obstetrics (FIGO) staging. Two tissue microarrays (TMAs) containing 436 ECs were used. Samples from the Institute of Basel were assembled into a new TMA (n = 248), while the Zurich-TMA was previously constructed (n = 188) [25]. Both TMAs included two tissue cores of each tumour. Patients with localised disease underwent hysterectomy and bilateral salpingo-oophorectomy (with/without pelvic and para-aortic lymphadenectomy). High-grade and myometrium invasive EC samples received adjuvant postoperative vaginal radiation and/or chemotherapy. Clinical outcome was available in 333 cases. Median follow-up was 42 months (1–184 months). The study was approved by the Cantonal Ethics Committee of Basel and Zurich (KEK-ZH-NR: 2010-0358).

### 19q12 amplicon detection using a dual-colour ISH automated assay

Tissue microarrays were analysed for 19q12 amplification status using a DNA probe set (Ventana Medical Systems, Tucson, Arizona), by measuring the copy number ratio of the 19q12 amplicon to the chromosome 19. The 19q12 ISH probe (Ventana) is a 2,4-dinitrophenyl (DNP)-labelled DNA probe, free of repetitive DNA sequences that covers a ~560 kb span of chromosome 19q12 containing the *CCNE1* and *URI* coding sequences. Due to homology within the alpha-satellite sequences of chromosomes 1 and 5, specifically identifying chromosome 19 is difficult. Therefore, a second repeat-free DNA probe was developed to allow chromosome 19 copy number enumeration. This digoxigenin (DIG)-labelled probe hybridises to a span of ~600 kb within 19p13.2-19p13.3, including the coding sequences for the insulin receptor INSR. The dual-colour ISH assay was previously established and automated using the Ventana BenchMark XT platform [7]. Briefly, after deparaffinisation, the TMA slides were treated with cell conditioning 2 for four 12-min cycles followed by ISH protease 2 for 8 or 12 min. After co-denaturation, 19q12 DNP and INSR DIG probes were hybridised at 51°C for 4 hours and washed three times at 68°C for 8 minutes/wash. The 19q12 DNP and INSR DIG signals were detected using Ventana ultraView SISH DNP and Ventana ultraView RED ISH DIG detection kits, respectively. Tumour samples were counterstained with haematoxylin II and bluing reagent.

Before CN enumeration and further analysis, samples were evaluated for acceptable staining based on the presence of appropriate signals in normal cells and adequate signal-to-background strength. Samples that failed to meet these criteria were rejected. After identifying regions for analysis, 19q12 and INSR signal numbers within 50 representative cells were recorded. In TMA cases containing small tumour tissue amounts, the total CN from 50 nuclei was obtained from multiple cores. Amplification was defined as the ratio of the average number of 19q12 copies to the average number of INSR copies/cell. Both parameters are detectable on one slide and appear as black (19q12) and red (INSR on chr19) dots. A sample was considered amplified if 19q12/INSR ratio was ≥2.0 [7, 15].

### Cyclin E1, URI and p53 immunohistochemistry (IHC)

TMA slides were incubated with a monoclonal antibody against Cyclin E1 (clone HE12, Santa Cruz Biotechnology, CA). Nuclear expression was scored according to the intensity of the immunostaining (0-no staining; 1-weak; 2-moderate; 3-strong) and the percentage of positive tumour cells. Afterwards, the H-score was calculated (ranging from 0–300) [7, 21]. A rabbit monoclonal antibody specific for URI (1-21, Ventana) was applied to TMAs and cytoplasmic expression was scored as above. The EC samples were investigated for p53 expression by immunohistochemistry (Dako, M7001) [26]. Tumour p53 expression strength was scored according to p53-positive cell numbers: 0%, 1%–75% or >75% (WHO Classification of Tumours of Female Reproductive Organs 2014).

### OncoScan CN assay

To independently confirm CN results from 19q12 ISH, we performed the OncoScan assay (Affymetrix, Inc) based on Molecular Inversion Probe (MIP) technology to analyse whole-genome CN variations (CNVs) in a 21 EC sample subset. The 19q12 ISH amplification status ranged from high CN in five EC samples (19q12/Chr19 ratio >3) to low amplification in 12 (19q12/Chr19 ratio 2–3) and no amplification in four (19q12/Chr19 ratio <2). For the OncoScan assay, formalin-fixed and paraffin-embedded (FFPE) cancer tissue blocks from 1985–1995 were punched and the removed sections (3–5 cylinders, 0.6-mm diameter) deparaffinised with xylene. After ethanol washing, genomic DNA was extracted with the DNeasy Blood and Tissue Kit (Qiagen #69504) following the manufacturer's protocol. The double-strand DNA concentration (dsDNA) was determined using the fluorescence-based Qubit dsDNA HS Assay Kit. The samples were further processed by IMGM Laboratories GmbH (Martinsried, Germany) for CNV determination according to the Affymetrix OncoScan FFPE assay recommended protocol.

Briefly, the assay uses locus-specific molecular inversion probes (MIPs) optimised for highly degraded FFPE samples with a 40-bp probe interrogation site. MIPs were hybridised to complementary DNA fragments leaving a gap at the SNP position of interest. The MIPs were circularised by adding a gap-filling enzyme, ligase and nucleotide complementary to the locus being interrogated. The gap-filling reaction was performed in separate steps for A/T and G/C nucleotides to properly distinguish between signals. After linear DNA fragment digestion (including gDNA and non-filled probes), the circularised probes were cleaved by HaeIII and amplified by PCR using common primers. The resulting probes (50 and 70 bp) were biotinylated and hybridised to separate OncoScan microarrays for A/T and G/C signals/sample. Signal strength gives information on the initial CN in a region of interest; SNPs help distinguish alleles. After hybridisation, microarrays were scanned with a 3000-7G GeneChip scanner (Affymetrix) and image files processed to yield signal intensity (.cel-) files. Cel files were then converted to .OSCHP files using OncoScan Console 1.3 (Affymetrix, Inc.), yielding normalised log intensity ratios (sample/reference), B-allele frequencies and a set of metrics for quality control, including a median of absolute pair-wise difference (MAPD), normal diploid SNP QC (ndSNPQC) or normal diploid waviness standard deviation (ndWavinessSD). After quality checking, samples were categorised as high, normal or low CN based on the log intensity ratio using the TuScan algorithm as implemented in Nexus Express Software for OncoScan 3.0.1 (Biodiscovery, Inc. 2014, CA, USA).

### Image digitisation

The bright field and fluorescence slide scanner Axio Scan.Z1 (Carl Zeiss, Jena, Germany) was used to digitise tissue cores at a resolution of ×40 (0.11 μm/pixel), according to the manufacturer's instructions.

### Image-based computational workflow (ISHProfiler)

Tissue cores were digitised and pre-processed (white balancing, deconvolution and contrast modification) using the scanner's default auto-correction settings. Images were then resized by bicubic interpolation to 4096 × 4096 pixels for efficient tiling (4096 = 212). These modified images served as input data for the computational workflow ISHProfiler. The computational workflow was implemented in MATLAB (R2014b) and tested on MacPro (2014). MATLAB built-in functions for the circular Hough transform (imfindcircles) and ROC analysis (perfcurve) were used. The software package LIBSVM (version 3.18) was used to train, validate and test support vector machine (SVM) models on the data.

### Statistics

Statistical analyses were conducted using IBM SPSS software (version 22). Associations between 19q12 amplification status and pathological parameters and Cyclin E1, URI and p53 expression levels were assessed by Pearson's chi-square or Fisher's exact test (two-sided). Differences in overall survival were determined by Kaplan–Meier analysis, and statistical significance was calculated using the log-rank test. Univariate and multivariate survival analyses were performed using the COX model of proportional hazards. *P* ≤ 0.05 was considered statistically significant. Pearson correlation analysis was conducted using R [R Core Team (2013). R: A language and environment for statistical computing. R Foundation for Statistical Computing, Vienna, Austria. ISBN 3-900051-07-0, URL https://www.r-project.org.

## SUPPLEMENTARY MATERIALS FIGURES



## References

[1] Bokhman JV (1983). Two pathogenetic types of endometrial carcinoma. Gynecol Oncol.

[2] Kandoth C, Schultz N, Cherniack AD, Akbani R, Liu Y, Shen H, Robertson AG, Pashtan I, Shen R, Benz CC, Yau C, Laird PW, Ding L, Zhang W, Mills GB, Cancer Genome Atlas Research N (2013). Integrated genomic characterization of endometrial carcinoma. Nature.

[3] Kuhn E, Wu RC, Guan B, Wu G, Zhang J, Wang Y, Song L, Yuan X, Wei L, Roden RB, Kuo KT, Nakayama K, Clarke B, Shaw P, Olvera N, Kurman RJ (2012). Identification of molecular pathway aberrations in uterine serous carcinoma by genome-wide analyses. Journal of the National Cancer Institute.

[4] Kuhn E, Bahadirli-Talbott A, Shih Ie M (2014). Frequent CCNE1 amplification in endometrial intraepithelial carcinoma and uterine serous carcinoma. Mod Pathol.

[5] Davis SJ, Sheppard KE, Pearson RB, Campbell IG, Gorringe KL, Simpson KJ (2013). Functional analysis of genes in regions commonly amplified in high-grade serous and endometrioid ovarian cancer. Clin Cancer Res.

[6] Theurillat JP, Metzler SC, Henzi N, Djouder N, Helbling M, Zimmermann AK, Jacob F, Soltermann A, Caduff R, Heinzelmann-Schwarz V, Moch H, Krek W (2011). URI is an oncogene amplified in ovarian cancer cells and is required for their survival. Cancer Cell.

[7] Noske A, Henricksen LA, LaFleur B, Zimmermann A, Tubbs A, Singh S, Storz M, Fink D, Moch H (2014). Characterization of the 19q12 amplification including CCNE1 and URI in different epithelial ovarian cancer subtypes. Experimental and molecular pathology.

[8] Gstaiger M, Luke B, Hess D, Oakeley EJ, Wirbelauer C, Blondel M, Vigneron M, Peter M, Krek W (2003). Control of nutrient-sensitive transcription programs by the unconventional prefoldin URI. Science.

[9] Galaal K, M Al Moundhri, Bryant A, Lopes AD, Lawrie TA (2014). Adjuvant chemotherapy for advanced endometrial cancer. Cochrane Database Syst Rev.

[10] Moxley KM, McMeekin DS (2010). Endometrial carcinoma: a review of chemotherapy, drug resistance, and the search for new agents. Oncologist.

[11] Etemadmoghadam D, deFazio A, Beroukhim R, Mermel C, George J, Getz G, Tothill R, Okamoto A, Raeder MB, Harnett P, Lade S, Akslen LA, Tinker AV, Locandro B, Alsop K, Chiew YE (2009). Integrated genome-wide DNA copy number and expression analysis identifies distinct mechanisms of primary chemoresistance in ovarian carcinomas. Clin Cancer Res.

[12] Patch AM, Christie EL, Etemadmoghadam D, Garsed DW, George J, Fereday S, Nones K, Cowin P, Alsop K, Bailey PJ, Kassahn KS, Newell F, Quinn MC, Kazakoff S, Quek K, Wilhelm-Benartzi C (2015). Whole-genome characterization of chemoresistant ovarian cancer. Nature.

[13] Lapenna S, Giordano A (2009). Cell cycle kinases as therapeutic targets for cancer. Nat Rev Drug Discov.

[14] Cicenas J, Valius M (2011). The CDK inhibitors in cancer research and therapy. J Cancer Res Clin Oncol.

[15] Kuhn E, Bahadirli-Talbott A, Shih IM (2013). Frequent CCNE1 amplification in endometrial intraepithelial carcinoma and uterine serous carcinoma. Mod Pathol.

[16] Zhong Q, Ruschoff JH, Guo T, Gabrani M, Schuffler PJ, Rechsteiner M, Liu Y, Fuchs TJ, Rupp NJ, Fankhauser C, Buhmann JM, Perner S, Poyet C, Blattner M, Soldini D, Moch H (2016). Image-based computational quantification and visualization of genetic alterations and tumour heterogeneity. Sci Rep.

[17] Cancer Genome Atlas Research N (2011). Integrated genomic analyses of ovarian carcinoma. Nature.

[18] Etemadmoghadam D, George J, Cowin PA, Cullinane C, Kansara M, Gorringe KL, Smyth GK, Bowtell DD, Australian Ovarian Cancer Study Group (2010). Amplicon-dependent CCNE1 expression is critical for clonogenic survival after cisplatin treatment and is correlated with 20q11 gain in ovarian cancer. PLoS One.

[19] Natrajan R, Mackay A, Wilkerson PM, Lambros MB, Wetterskog D, Arnedos M, Shiu KK, Geyer FC, Langerod A, Kreike B, Reyal F, Horlings HM, van de Vijver MJ, Palacios J, Weigelt B, Reis-Filho JS (2012). Functional characterization of the 19q12 amplicon in grade III breast cancers. Breast Cancer Res.

[20] Karst AM, Jones PM, Vena N, Ligon AH, Liu JF, Hirsch MS, Etemadmoghadam D, Bowtell DD, Drapkin R (2014). Cyclin e1 deregulation occurs early in secretory cell transformation to promote formation of fallopian tube-derived high-grade serous ovarian cancers. Cancer Res.

[21] Nakayama N, Nakayama K, Shamima Y, Ishikawa M, Katagiri A, Iida K, Miyazaki K (2010). Gene amplification CCNE1 is related to poor survival and potential therapeutic target in ovarian cancer. Cancer.

[22] Pils D, Bachmayr-Heyda A, Auer K, Svoboda M, Auner V, Hager G, Obermayr E, Reiner A, Reinthaller A, Speiser P, Braicu I, Sehouli J, Lambrechts S, Vergote I, Mahner S, Berger A (2014). Cyclin E1 (CCNE1) as independent positive prognostic factor in advanced stage serous ovarian cancer patients - a study of the OVCAD consortium. Eur J Cancer.

[23] Etemadmoghadam D, Weir BA, Au-Yeung G, Alsop K, Mitchell G, George J, Davis S, D'Andrea AD, Simpson K, Hahn WC, Bowtell DD, Australian Ovarian Cancer Study Group (2013). Synthetic lethality between CCNE1 amplification and loss of BRCA1. Proc Natl Acad Sci U S A.

[24] Etemadmoghadam D, Au-Yeung G, Wall M, Mitchell C, Kansara M, Loehrer E, Batzios C, George J, Ftouni S, Weir BA, Carter S, Gresshoff I, Mileshkin L, Rischin D, Hahn WC, Waring PM (2013). Resistance to CDK2 inhibitors is associated with selection of polyploid cells in CCNE1-amplified ovarian cancer. Clin Cancer Res.

[25] Ikenberg K, Valtcheva N, Brandt S, Zhong Q, Wong CE, Noske A, Rechsteiner M, Rueschoff JH, Caduff R, Dellas A, Obermann E, Fink D, Fuchs T, Krek W, Moch H, Frew IJ (2014). KPNA2 is overexpressed in human and mouse endometrial cancers and promotes cellular proliferation. The Journal of pathology.

[26] Wild PJ, Ikenberg K, Fuchs TJ, Rechsteiner M, Georgiev S, Fankhauser N, Noske A, Roessle M, Caduff R, Dellas A, Fink D, Moch H, Krek W, Frew IJ (2012). p53 suppresses type II endometrial carcinomas in mice and governs endometrial tumour aggressiveness in humans. EMBO Mol Med.

