# Supplementary material for: Detection of CCNE1/URI (19q12) amplification by in situ hybridisation is common in high grade and type II endometrial cancer
Source: Oncotarget. 2016 Aug 25;8(9):14794–805. doi: 10.18632/oncotarget.11605 (PMC5362444; doi:10.18632/oncotarget.11605)
Supplement: Supplementary file 1 [file oncotarget-08-14794-s001.pdf]

# Detection of *CCNE1/URI* (19q12) amplification by *in situ* hybridisation is common in high grade and type II endometrial cancer

## SUPPLEMENTARY FIGURES

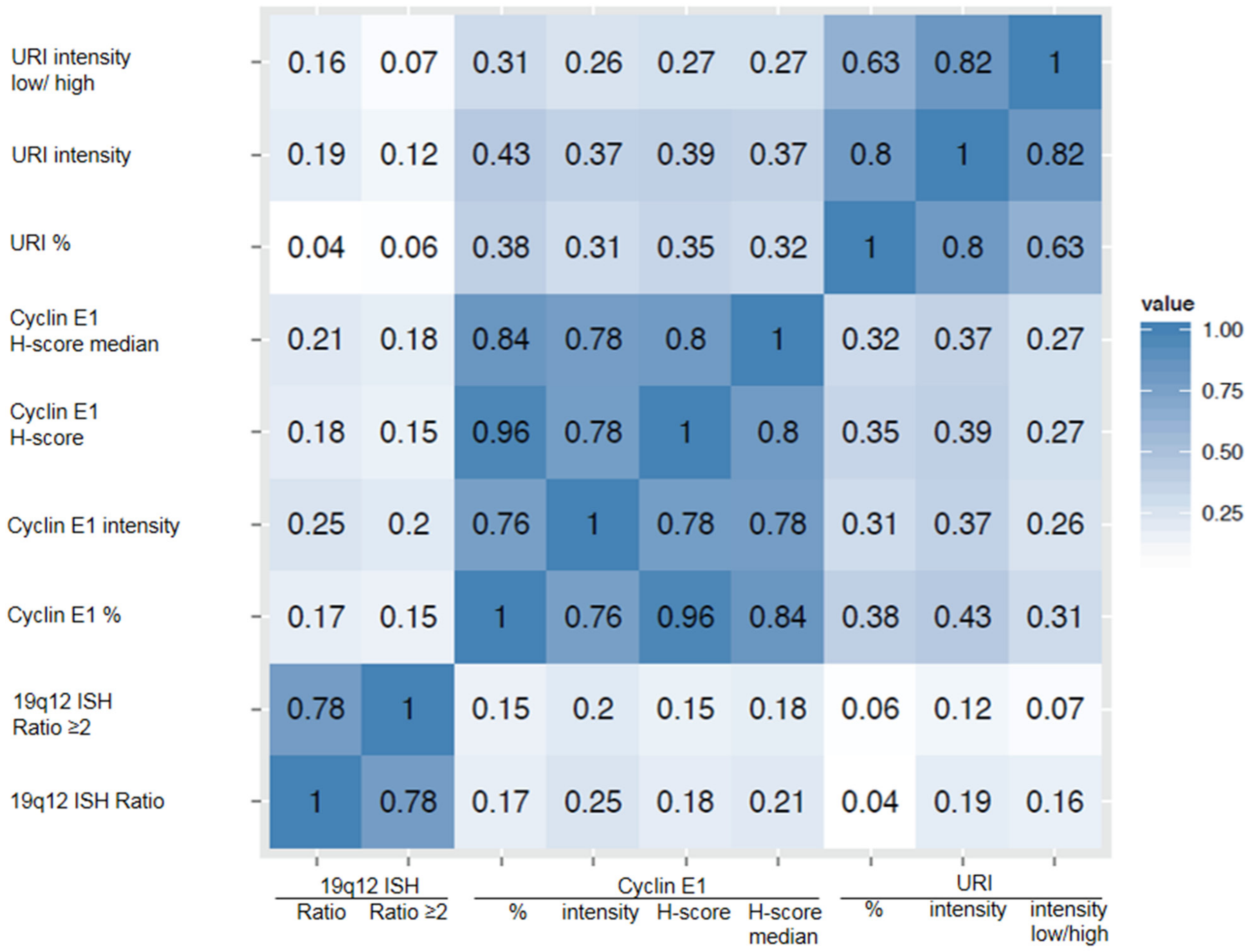


**Supplementary Figure S1: Correlation plot illustrating the relationship between 19q12 ISH status and IHC expression of Cyclin E1 and URI.** 19q12 ISH is indicated with the basic ratio (19q12/INSR) and cut-off value (ratio ≥2). IHC of both markers is given according to the intensity and percentage of positive tumor cells (%). For URI, the staining intensity was classified into low and high (URI intensity grouped). The IHC of Cyclin E1 was further categorized by using the H-score and divided in low and high according to the median H-score. The values of correlation vary between 0 and 0.5 indicating weak relationships between the variables.

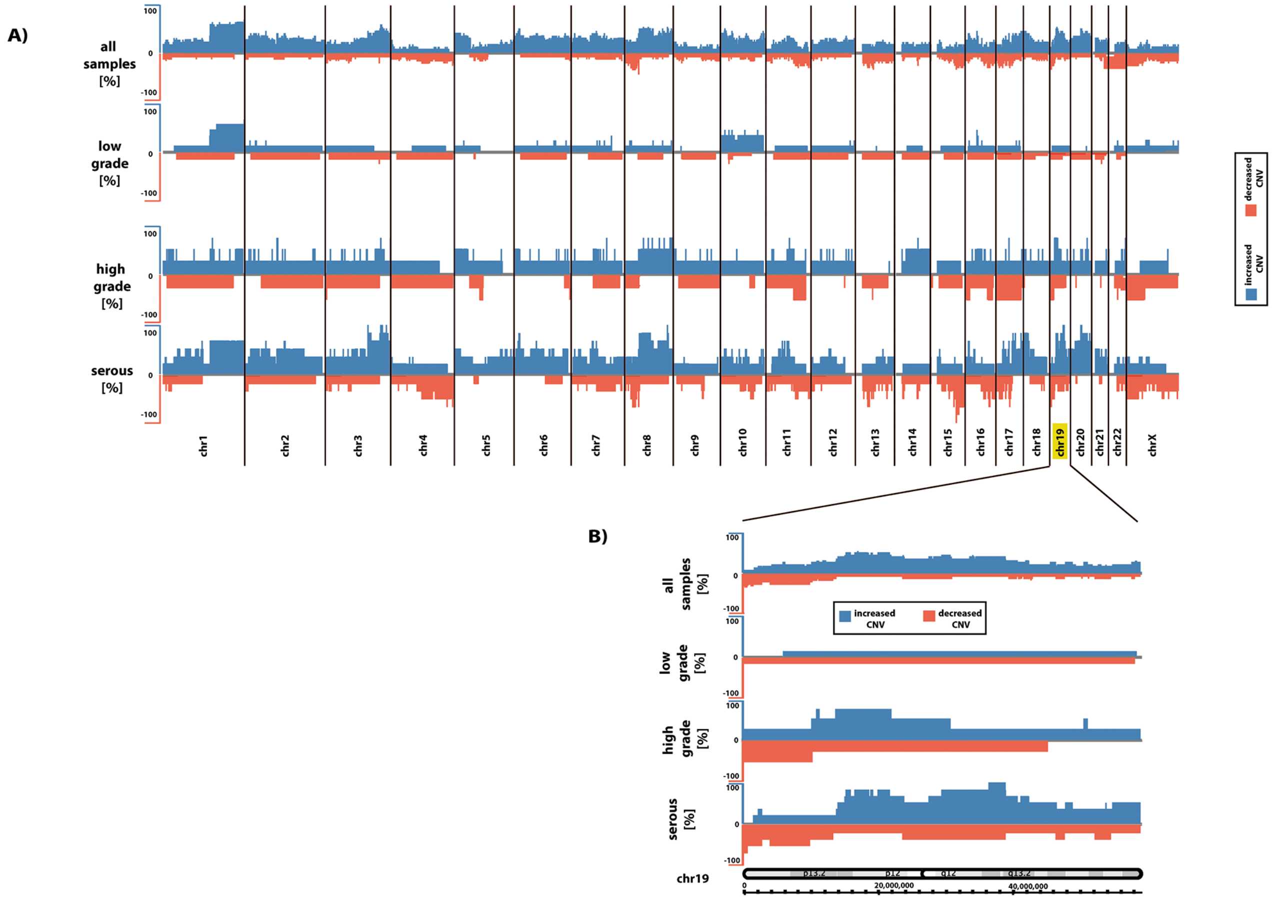

**Supplementary Figure S2: Genome-wide copy number (CN) profiling of low-grade endometrioid EC, high-grade endometrioid EC, serous EC and all subtypes combined.** Genome-wide copy numbers were detected using OncoScan assay for each individual sample. For each subtype and for all samples, the respective percentages of samples showing increased CN (blue) or decreased CN (red) are shown throughout each chromosome (A) and in detail for chromosome 19 (B).
